# An educational intervention to promote appropriate antibiotic use for acute respiratory infections in a district in Egypt- pilot study

**DOI:** 10.1186/s12889-019-6779-0

**Published:** 2019-05-10

**Authors:** Amr Kandeel, Danielle L. Palms, Salma Afifi, Yasser Kandeel, Ahmed Etman, Lauri A. Hicks, Maha Talaat

**Affiliations:** 1 Ministry of Health, Cairo, Egypt; 2 Centers for Disease Control and Prevention, Atlanta, GA 30329 USA; 3 Global Disease Detection Center, US CDC, Cairo, Egypt

**Keywords:** Antibiotics, Acute respiratory infection, Cold, Bronchitis, Sinusitis, Egypt, Global health security

## Abstract

**Background:**

Antibiotic overuse is the most important modifiable factor contributing to antibiotic resistance. We conducted an educational campaign in Minya, Egypt targeting prescribers and the public through communications focused on appropriate antibiotic use for acute respiratory infections (ARIs).

**Methods:**

The entire population of Minya was targeted by the campaign. Physicians and pharmacists were invited to participate in the pre-intervention assessments. Acute care hospitals and a sample of primary healthcare centers in Minya were randomly selected for a pre-intervention survey and all patients exiting outpatient clinics on the day of the survey were invited to participate. The same survey methodology was conducted for the post-intervention assessments. Descriptive comparisons were made through three assessments conducted pre- and post-intervention. We quantitated antibiotic prescribing through a survey administered to patients with an ARI exiting outpatient clinics. Additionally, physicians, pharmacists, and patients were interviewed regarding their attitudes and beliefs towards antibiotic prescribing. Finally, physicians were tested on three clinical scenarios (cold, bronchitis, and sinusitis) to measure their knowledge on antibiotic use.

**Results:**

Post-intervention patient exit surveys revealed a 23.1% decrease in antibiotic prescribing for ARIs in this population (83.7 to 64.4%) and physicians and pharmacists self-reported less frequently prescribing antibiotics for ARIs on their follow-up surveys. We also found an increase in correct responses to the clinical scenarios and in attitude and belief scores for physicians, pharmacists, and patients regarding antibiotic use in the post-intervention sample.

**Conclusions:**

Overall, the samples surveyed after the community-based educational campaign reported a lower frequency of antibiotic prescribing and improved knowledge and attitudes regarding antibiotic misuse compared to the samples surveyed before the campaign. Ongoing interventions educating providers and patients are needed to decrease antibiotic misuse and reduce the spread of antibiotic resistance in Egypt.

## Background

Antibiotic resistance is a growing public health threat worldwide and a major threat to global health security. Increasing antibiotic resistance is a consequence of selective pressures created by use of antibiotics. Respiratory infections are the leading reason for antibiotic prescriptions in both the adult and pediatric populations. Overuse of antibiotics, such as taking antibiotics when they aren’t needed for viral infections, is a driver of antibiotic resistance, which is a serious problem for public health, individual patients, and healthcare systems. Antibiotics are most frequently prescribed for management of acute respiratory tract infections (ARIs), including rhinosinusitis, pharyngitis, bronchitis, otitis media, and nonspecific ARIs [1]. Published data from the pre-intervention study revealed that 82% of pediatric visits for ARIs and 85% of adult visits for ARIs resulted in an antibiotic prescription in Minya District, Egypt. The pre-intervention data found particularly high antibiotic prescribing for infections that do not warrant antibiotics, including 53% prescribing among pediatric common cold visits and 94% prescribing among adult bronchitis visits [2].

Numerous interventions to improve antibiotic prescribing practices have been reported from various countries with varying results [3–5]. No single intervention appears to have superior efficacy, but combinations of interventions are typically more effective, [6, 7] and strategies that target health care professionals and patients (or parents of young children) have achieved success at reducing antibiotic prescriptions for ARIs. Most of these interventions have focused on educational sessions and materials for health care professionals and patients in urban and suburban settings [8–10].

Recent studies in Egypt have shown that antibiotics are used extensively for treatment of ARIs [11, 12]. Therefore, an intervention to improve antibiotic use was piloted to assess the potential usefulness for future widespread implementation. The aim of this pilot was to describe antibiotic prescribing practices for ARIs and knowledge and attitudes of physicians, pharmacists, and patients before and after a multi-dimensional behavior change strategy.

## Methods

### Design and setting

Baseline surveys were conducted in Minya District in Upper Egypt from May–July 2011 at four acute care hospitals and randomly selected 41 primary healthcare units (PHU) (representing 50% of PHUs in Minya). All patients that presented to these facilities during the day of the visit were invited to participate in the survey. Patients with an ARI were interviewed as they were exiting outpatient clinics (internal medicine, pediatrics, chest, general medicine, otolaryngology (ENT), or general outpatient clinics) to estimate the frequency of antibiotic prescribing for ARIs. A post-intervention survey was repeated using the same sampling strategy and number of health facilities, from October–December, 2012. Interviews were conducted with adult patients (age 18 years or older) or with a parent or caretaker of pediatric patients (less than 18 years of age). The detailed methods and results of the baseline patient exit survey are published elsewhere [2]. The intervention was implemented throughout the district during August – December 2011 and the same survey methodology was repeated in the post-intervention period from October – December 2012 in the same hospitals and different random sample of PHUs to measure the percent change in physician antibiotic prescribing for patients with ARIs visiting ambulatory care. Therefore, the random samples were from the same larger population.

### Physician and pharmacist knowledge, attitudes, and practices survey

This survey targeted physicians and pharmacists to measure their attitudes and practices related to treating patients with ARIs [13]. All physicians with the following specialties: general practice, chest medicine, internal medicine, pediatrics, and otolaryngology and pharmacists employed at primary healthcare centers, hospitals, or private pharmacies in Minya District were invited to participate in the surveys. Both physicians and pharmacists were interviewed pre-intervention using a standardized questionnaire which collected demographic information, self-reported prescribing practices for ARIs, and a set of variables to measure their beliefs and attitudes regarding antibiotic prescribing related to treatment of ARIs, as well as their attitudes towards antibiotic resistance. In the post-intervention period, physicians and pharmacists in Minya District using the same inclusion criteria were invited to participate in the post-intervention survey. Therefore, all physicians and pharmacists from the same population were invited to be included in the pre- and post-intervention samples. The mean of the attitude scores reflecting the judicious use of antibiotics for physicians and pharmacists and the self-reported antibiotic prescribing practices for ARIs for physicians and pharmacists were measured.

In addition, physicians were presented three clinical scenarios that did not warrant antibiotic therapy (cold, bronchitis, and sinusitis), and asked how they would treat these patients. The knowledge of the physicians regarding treatment choices in response to these ARIs was measured.

### Public beliefs and attitudes towards appropriate use of antibiotics

The same data collection tool used to measure physician and pharmacist beliefs and attitudes about antibiotic use and resistance was used for patients who participated in the patient exit interviews in the pre- and post-intervention periods. The same scoring procedures were also used to measure the beliefs and attitudes before and after the intervention.

Sample size calculations were estimated in preparation for the pre-intervention surveys assuming that physicians prescribed antibiotics for 60% of ARI visits and that antibiotic prescribing for ARIs would be reduced by 20% post-intervention, with a significance level of 95% and a power of 90%. The details of the sample size calculations are described elsewhere [2, 13].

### Communication campaign intervention

An intensive campaign to promote appropriate antibiotic prescription was launched in Minya District from August to December 2011. The aim of the campaign was to raise the awareness of physicians, pharmacists, and the general public in the district regarding the importance of rational antibiotic prescribing for ARIs. The primary audience included all physician specialties that might be involved with treatment of patients with ARIs (internal medicine, pediatrics, chest, general medicine, ENT, or general practitioners), either in the government or private sector. In addition, pharmacists and their assistants in the public and private sector were also targeted. The campaign targeted all physicians, pharmacists, and the general public in Minya. Random samples of patients from the Minya District were interviewed pre- and post-intervention and physicians and pharmacists were invited to participate in the pre- and post-intervention surveys as described above in order to describe the reach of the campaign throughout the entire district.

A social media campaign targeting educated youth was implemented using Facebook and the YouTube channel. A page named after the campaign slogan “Be wise in using antibiotics” was added on Facebook and YouTube. Three moderators were leading a discussion on appropriate antibiotic use in Arabic. Electronic copies of the informational, educational, and communication materials were uploaded on the Facebook page. Videos produced by CDC’s Get Smart campaign were translated and subtitles were added in Arabic after obtaining CDC approval. These videos were uploaded to the YouTube channel. Animated videos were also produced to act as viral videos to bring users to the campaign pages.

#### Messages

Multiple focus group discussions were conducted with physicians, pharmacists, non-governmental organizations, and the general public in order to define the key messages and potential communication channels. The message themes for physicians and pharmacists were mainly to improve knowledge regarding appropriate antibiotic prescribing. Specific messages were focused on avoiding antibiotic prescribing for viral ARIs.

Informational, educational, and communication materials were developed to target three groups (physicians, pharmacists, and the general public). For physicians and pharmacists, Centers for Disease Control and Prevention fact sheets on ARI management guidelines and algorithms were revised and updated by an international consultant and printed with different color codes for each type of respiratory infection (e.g. common cold, bronchitis, and sinusitis). A notebook was designed and printed including the fact sheets as page separators. Reminder messages were printed on 12-page desktop calendars including the principles of antibiotic prescriptions for ARIs. To improve awareness on antibiotic resistance, a promotion folder was prepared which delivered information on the burden of antibiotic resistance and its consequences. For the general public, posters communicating specific simple messages to decrease their demand for antibiotics were displayed in doctor clinic waiting areas, pharmacies, community gathering areas, universities, and schools.

#### Training of physicians and pharmacists

A 5-day training course by an international infectious disease consultant was held to educate participants on appropriate management of ARIs. The workshop targeted all clinical specialties and primary health care doctors of Minya District that treat ARIs (internal medicine, chest, tropical, ENT, and pediatrics). Educational sessions were also provided to pharmacists and focused mainly on the difference between bacterial and viral infections, and the contribution of antibiotic overuse to the development of antibiotic resistance. A total of 315 physicians and pharmacists attended the training workshops, which represented 70% of physicians and pharmacists employed in the government sector. To ensure district-wide coverage of scientific information, three senior medical doctors and 13 pharmacists that attended the training by the international consultant and showed special interest and skills were recruited to conduct academic detailing and visit and educate private physicians and pharmacists that did not attend the training. They used the educational materials to disseminate specific campaign messages to doctors and pharmacists.

#### Analysis

Percent change was calculated to descriptively compare antibiotic prescribing for pediatric and adult patients before and after the intervention. To describe the attitudes and beliefs of the patients towards antibiotic use, a scoring system described previously by Taylor et al., 2003 [14] was used. Responses to attitude questions were transformed into an ordinal scale with scores ranging from 0 to 5. For the questions where agreement was indicative of an attitude supportive of proper use of antibiotics, a score of 5 was applied to the response of “strongly agree” and 0 for “strongly disagree”. On the other hand, for questions where disagreement supported proper use of antibiotics, a score of 5 was applied to the answer “strongly disagree” and 0 for “strongly agree”. Means of scores were descriptively compared before and after the intervention. All analyses were conducted using Epi Info 7.

## Results

### Frequency of antibiotic prescribing for ARI visits

Table 1 describes the numbers of the various target groups engaged in both the pre- and post-intervention surveys. Table 2 presents the prescribing practices for ARIs among physicians in the pre- and post-intervention surveys. There was a 25% decrease overall in antibiotic prescribing post-intervention for children from 82.1 to 61.5%; the largest improvements in prescribing in these patients were for ear infection and common cold. There was a 22% decrease overall in antibiotic prescribing for adults from 86.7 to 67.9%, which was driven by large changes in prescribing for patients with ear infections and bronchitis. (Table 2).

**Table 1:** Demographic characteristics of study population in pre and post intervention surveys, Minya, 2012

Characteristic	Pre-intervention		Post-intervention	
	N	%	N	%
Adult patients^a^ (≥18 years old)	*N* = 113		*N* = 277	
Age in years (mean ± SD)	37.2 ± 16		36.0 ± 17	
Males	42	37.2	111	40.1
Education				
Illiterate	50	44.2	169	61.0
Primary education	22	19.5	39	14.1
Secondary	30	26.5	54	19.5
University	11	9.7	15	5.4
Pediatric patients^a^ (< 18 years old)	*N* = 218		*N* = 330	
Age in years (mean ± SD)	4.9 ± 4		4.4 ± 3	
Males	109	50.0	188	57.0
Education				
Illiterate (Parents)	90	41.3	213	64.5
Primary education	48	22.0	39	11.8
Secondary	73	33.5	56	17.0
University	7	3.2	22	6.7
Clinicians	*N* = 237		*N* = 289	
Age in years (mean ± SD)	41.2 ± 11		39.3 ± 11	
Males	134	56.5	131	45.3
Specialty				
General Practice/ Family medicine	65	27.4	104	36.0
Internal Medicine	63	26.6	71	24.6
Pediatrician	59	24.9	71	24.6
Chest	21	8.9	14	4.8
Tropical Medicine	15	6.3	11	3.8
ENT	14	5.9	18	6.2
Type of work facility				
Governmental health facility	214	90.3	199	68.9
Governmental + private health facility	23	9.7	90	31.1
Pharmacists	*N* = 483		*N* = 596	
Type of Pharmacy				
Private	345	71.4	397	66.6
Hospital	97	20.1	190	31.9
Primary Health Unit	41	8.5	9	1.5
Respondent				
Pharmacist	307	63.6	466	78.2
Assistant	176	36.4	130	21.8
Age in years (mean ± SD)	30.6 ± 10		29.7 ± 9	
Males	272	56.3	296	49.7
Mean No. of years of experience	8.3 ± 9		7.0 ± 8	
Mean No. of customers/day	79.5 ± 104		67.2 ± 85	

^a^Patients with pneumonia excluded

**Table 2:** Antibiotic prescribing frequency for acute respiratory infections according to clinical diagnosis pre- and post-intervention, Minya, 2012

Diagnosis	Pre-intervention			Post-intervention		
	Encounters reviewed	Encounters including antibiotic prescription		Encounters reviewed	Encounters including antibiotic prescription	
	N	N	%	N	N	%
Pediatric						
Ear infection	2	2	100.0	5	3	60.0
Tonsillitis	35	34	97.1	35	34	97.1
Pharyngitis	53	51	96.2	36	30	83.3
Sinusitis	7	6	85.7	14	14	100.0
Bronchitis	78	63	80.8	97	68	70.1
Common cold	43	23	53.5	143	54	37.8
Total pediatric	218	179	82.1	330	203	61.5
Adults						
Ear infection	4	4	100.0	2	1	50.0
Tonsillitis	10	10	100.0	17	17	100.0
Pharyngitis	27	26	96.3	61	55	90.2
Sinusitis	10	8	80.0	10	10	100.0
Bronchitis	35	33	94.3	77	49	63.6
Common cold	27	17	63.0	110	56	50.9
Total adults	113	98	86.7	277	188	67.9
Grand total	331	277	83.7	607	391	64.4

### Physician and pharmacist belief and attitude scores towards use of antibiotics

The mean knowledge and attitude scores supporting the judicious use of antibiotics improved after comparing responses pre- and post-intervention for both physicians and pharmacists (from 3.8 ± 0.5 to 4.0 ± 0.7 for physicians and from 3.3 ± 0.9 to 4.0 ± 1.2 for pharmacists). (Table 3) Both groups had improved attitude scores regarding reducing antibiotic prescribing for cold symptoms that last more than 5 days; limiting the use of antibiotics to preserve their effectiveness; not giving antibiotics based on nasal discharge appearance; and not giving antibiotics to help cold symptoms clear up more quickly or to treat colds. Attitudes related to not overusing antibiotics to prevent resistant bacteria improved among pharmacists, while not prescribing antibiotics to patients with colds to prevent infection improved among physicians. (Table 3).

**Table 3:** Belief and attitude scores regarding the judicious use of antibiotics among physicians and pharmacists pre- and post-intervention^a^

	Physicians		Pharmacists	
	Pre-intervention *n* = 237	Post-intervention *n* = 289	Pre-intervention *n* = 483	Post-intervention *n* = 596
	Mean ± SD	Mean ± SD	Mean ± SD	Mean ± SD
Physicians should never prescribe antibiotics when they are unnecessary	4.8 ± 0.7	4.7 ± 0.7	4.5 ± 1.1	4.6 ± 1.0
Too many people are treated with antibiotics when not necessary	3.5 ± 1.5	3.3 ± 1.7	3.7 ± 1.4	3.8 ± 1.5
Overuse of antibiotics can make bacteria more resistant to antibiotics	4.6 ± 0.9	4.7 ± 1.0	3.9 ± 1.7	4.1 ± 1.5
Giving an antibiotic to a patient with cold symptoms can prevent an infection from occurring	3.9 ± 1.4	4.3 ± 1.2	3.5 ± 1.4	3.6 ± 1.6
It is worth trying an antibiotic when someone has cold symptoms for 5 days	3.9 ± 1.2	4.3 ± 1.1	2.8 ± 1.6	3.7 ± 1.5
Using antibiotics frequently doesn’t make them less effective	4.5 ± 1.2	4.7 ± 0.9	3.8 ± 1.6	4.4 ± 1.3
Treatment with antibiotics is necessary when nasal discharge turns from yellow to green in color	1.8 ± 1.2	3.2 ± 1.6	1.4 ± 1.2	3.0 ± 1.7
Antibiotics help cold symptoms clear up more quickly	4.3 ± 1.1	4.6 ± 0.9	3.4 ± 1.5	4.1 ± 1.4
Antibiotics are helpful in treating colds	4.2 ± 1.1	4.7 ± 0.7	3.2 ± 1.5	4.0 ± 1.4
Overall scores	3.8 ± 0.5	4.0 ± 0.7	3.3 ± 0.9	4.0 ± 1.2

^a^All responses ranged from “strongly disagree” to “strongly agree.” For questions where agreement supported proper antibiotic use, a response of “strongly agree” was scored a 5 and “strongly disagree” was scored a 0. For questions where disagreement supported proper antibiotic use, a response of “strongly disagree” was scored a 5 and “strongly agree” was scored a 0.

Table 4 presents the self-reported antibiotic prescribing practices for ARIs for physicians and pharmacists in the pre- and post-intervention periods. The percent of physicians who reported prescribing antibiotics most of the time for common cold, bronchitis, and sinusitis decreased after the intervention (from 9.3 to 2.1%, from 65.8 to 28.4%, and from 43.5 to 17.0%, respectively). There was a 31% decrease in pharmacists reporting prescribing antibiotics for an ARI (from 83.6 to 57.7%), a 57% decrease in pharmacists reporting recommending antibiotics for an ARI (from 57.8 to 24.8%), and a 59% decrease in pharmacists reporting prescribing antibiotic for common cold (from 67.5 to 28.0%) after the intervention. No data was available for pharmacists for treating bronchitis or sinusitis as they do not examine patients to confirm the diagnosis.

**Table 4:** Self-reported antibiotic prescribing practices for ARIs for physicians and pharmacists pre- and post-intervention

Prescribing practice	Physicians				Pharmacists			
	Pre-intervention (*n* = 237)		Post-intervention (*n* = 289)		Pre-intervention (*n* = 483)		Post-intervention (*n* = 596)	
	N	%	N	%	N	%	N	%
Prescribing antibiotics (pharmacists only)								
Prescribe antibiotics					404	83.6	344	57.7
Recommend antibiotics					279	57.8	148	24.8
Prescribing frequency								
Common cold								
Most times	22	9.3	6	2.1	44	9.1	17	2.9
Sometimes	128	54.0	97	33.6	282	58.4	150	25.2
Never	84	35.4	186	64.4	78	16.1	177	29.7
Bronchitis (physicians only)								
Most times	156	65.8	82	28.4				
Sometimes	71	30.0	179	61.9				
Never	6	2.5	25	8.7				
Sinusitis (physicians only)								
Most times	103	43.5	49	17.0				
Sometimes	117	49.4	183	63.3				
Never	12	5.1	52	18.0				

### Clinical scenarios

**Fig. 1:**
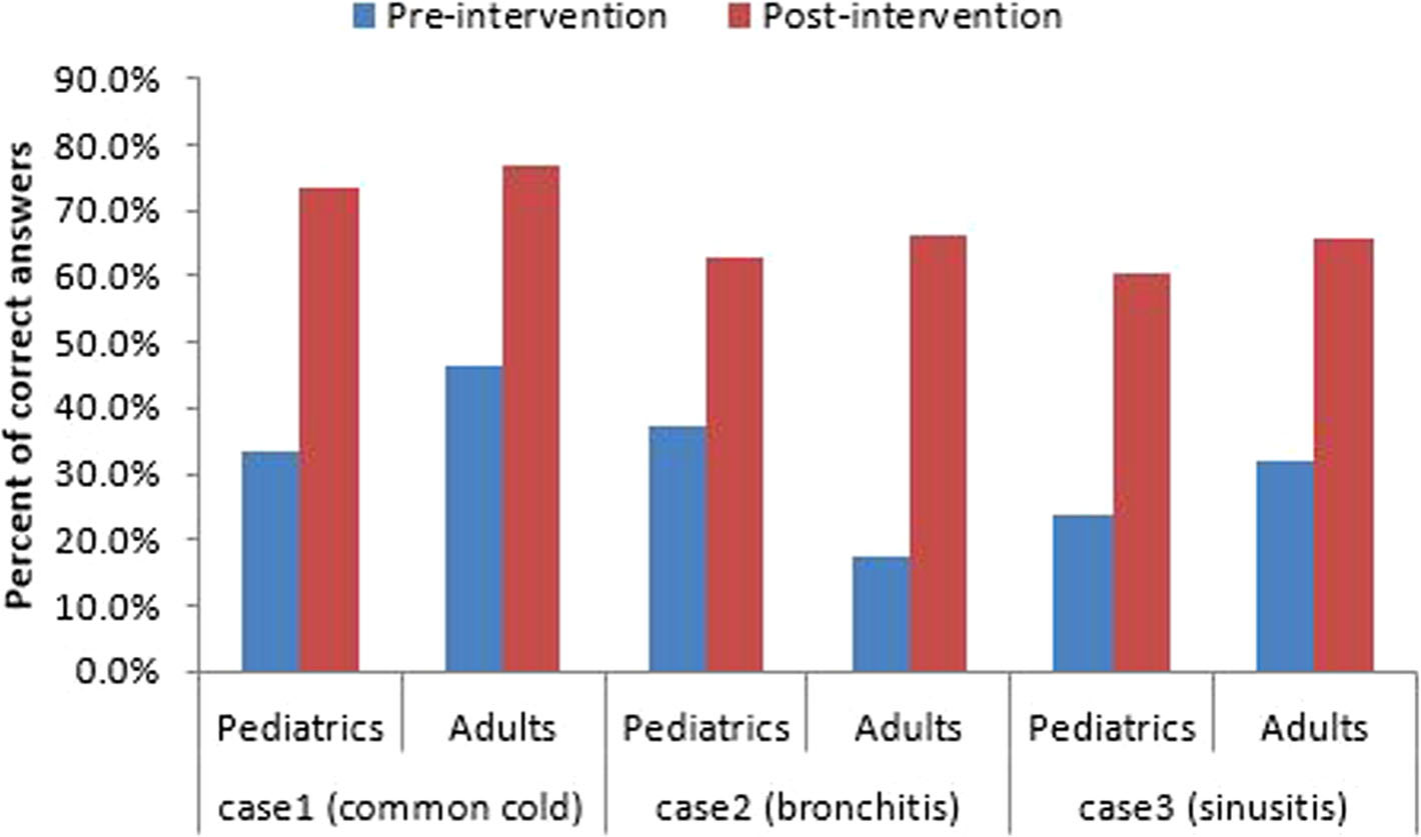
Physician treatment choices in response to pediatric and adult acute respiratory infection clinical scenarios, pre- and post-intervention

The percent of physicians who gave correct answers to the common cold, bronchitis, and sinusitis case scenarios increased in the post-intervention survey compared to the pre-intervention survey for both pediatric and adult case scenarios. The largest improvement was shown for the adult bronchitis case scenario where there was a 287% increase in correct responses (from 17.2 to 66.5%). (Fig. 1).

### Public belief and attitude scores towards the use of antibiotics

There was an overall increase (improvement) in the mean attitude scores for both groups of patients (parents of pediatrics and adults) between the pre-and post-intervention periods (Table 5). Both patient groups improved their mean scores regarding persuading a doctor to prescribe antibiotics from 4.1 pre-intervention to 4.4 after the intervention, indicating that they were less likely to persuade doctors to prescribe. Both caregivers and adult patients also better understood that many people are treated with antibiotics when they are not necessary (mean scores increased from 2.9 to 3.3 and from 2.7 to 3.4, respectively). Caregivers gained knowledge that treatment with antibiotics is unnecessary when nasal discharge turns from yellow to green in color (from 1.0 to 1.4), and adult patients better understood that overuse of antibiotics can make bacteria more resistant to antibiotics (from 1.6 to 2.1). Overall knowledge and attitude scores increased among caregivers and adult patients after the intervention (from 2.3 to 2.5 and from 2.4 to 2.6, respectively). (Table 5).

**Table 5:** Public belief and attitude scores towards judicious use of antibiotics, pre- and post-intervention, Minya, 2011^a^

	Parents of pediatric patients		Adult patients	
	Pre-intervention (*n* = 227)	Post-intervention (*n* = 343)	Pre-intervention (*n* = 123)	Post-intervention (*n* = 280)
	Mean ± SD	Mean ± SD	Mean ± SD	Mean ± SD
Healthcare provider should not prescribe antibiotics unnecessarily	4.1 ± 1.5	4.3 ± 1.3	4.0 ± 1.6	4.2 ± 1.4
You should not try to persuade a doctor to prescribe antibiotics	4.1 ± 1.5	4.4 ± 1.0	4.1 ± 1.6	4.4 ± 1.2
Too many people are treated with antibiotics when not necessary	2.9 ± 2.0	3.3 ± 1.9	2.7 ± 1.9	3.4 ± 1.9
Overuse of antibiotics can make bacteria more resistant to antibiotics	1.5 ± 1.9	1.8 ± 2.2	1.6 ± 2.1	2.1 ± 2.3
Giving an antibiotic to a patient with cold symptoms can prevent an infection from occurring	1.7 ± 1.9	1.2 ± 1.6	1.7 ± 1.9	1.2 ± 1.6
It is worth trying an antibiotic when someone has cold symptoms for 5 days	2.1 ± 1.8	2.0 ± 1.6	2.2 ± 1.8	1.9 ± 1.5
Treatment with antibiotics is necessary when nasal discharge turns from yellow to green in color	1.0 ± 1.6	1.4 ± 1.5	1.1 ± 1.6	1.1 ± 1.4
Antibiotics help cold symptoms clear up more quickly	1.8 ± 1.5	1.9 ± 1.7	2.0 ± 1.7	1.9 ± 1.7
Antibiotics are helpful in treating colds	1.9 ± 1.6	1.9 ± 1.7	2.2 ± 1.7	1.9 ± 1.7
Overall Scores	2.3 ± 0.9	2.5 ± 1.0	2.4 ± 0.9	2.6 ± 1.0

^a^All responses ranged from “strongly disagree” to “strongly agree.” For questions where agreement supported proper antibiotic use, a response of “strongly agree” was scored a 5 and “strongly disagree” was scored a 0. For questions where disagreement supported proper antibiotic use, a response of “strongly disagree” was scored a 5 and “strongly agree” was scored a 0.

## Discussion

Following an educational intervention, including a public education campaign, provider education, and academic detailing, to improve antibiotic use for ARIs in Minya District, Egypt there were reported improvements in antibiotic prescribing and knowledge. The results of the present study are consistent with evidence from similar settings including two previous studies in hospitals in Thailand that found overall improved antibiotic use and reduced incidence of inappropriate use following educational campaigns [15, 16]. Baseline surveys in Minya District, Egypt revealed a high frequency of antibiotic prescribing for ARIs and gaps in knowledge about appropriate antibiotic prescribing for various clinical scenarios and antibiotic resistance among patients, physicians, and pharmacists. In a setting where patients can purchase antibiotics without a physician prescription, this educational intervention aimed to increase awareness about appropriate use not only among physicians and pharmacists, but also among patients who can independently purchase antibiotics over the counter. This study is the first time an educational intervention aimed at improving antibiotic use was implemented and evaluated in Egypt.

The follow-up surveys of patients leaving outpatient visits for ARIs in Minya demonstrated a decrease in antibiotic prescribing after the educational intervention. Comparing survey results before and after the intervention, prescribing decreased in both pediatric and adult visits for ARI symptoms. Although this is the first study of its kind in Egypt, similar studies assessing antibiotic use interventions have been conducted in other countries. Two studies in the United States implementing educational interventions to address pediatric prescribing led to intervention-attributable declines in antibiotic prescription rates between 11 and 16% [8, 10]. Another US study focused on decreasing prescribing for ARIs among adults saw prescribing for acute bronchitis drop from 58 to 30% for those receiving a limited intervention and to 24% for those receiving a full intervention [9]. Although the current study revealed baseline prescribing rates were higher in Egypt compared to similar US studies, antibiotic prescribing for ARIs was decreased following an educational intervention in both studies.

The current intervention also demonstrated improved knowledge and attitudes regarding appropriate antibiotic use among physicians and pharmacists surveyed following the intervention compared to pre-intervention. A similar study in the United States found that after a statewide educational campaign, desired responses about antibiotic use in case scenarios for upper respiratory infection and bronchitis increased among clinicians [17]. Additionally, the current study found an improvement in mean belief and attitude scores among parents of pediatric patients and among adult patients after the educational campaign. These results were consistent with two previous studies including one in the US and one in Israel utilizing educational campaigns demonstrating improved knowledge about antibiotics and decreased desire for antibiotics for ARIs among patients and parents of pediatric patients [18, 19]. Therefore, the results of the present study add to the evidence that knowledge and behaviors regarding antibiotic use can improve in a variety of settings following low-cost educational interventions.

Based on data from this pilot, the health authorities in Minya District developed policies for appropriate antibiotic use for ARIs that were endorsed by local physicians, and included distribution of the policies and regular auditing and review of physician prescriptions. Additionally, the Ministry of Health and Population in Egypt is exploring feasible methods and approaches to expand the communication campaign in phases to more regions in Egypt using the model applied in Minya and the available information, education, and communication materials.

This study is subject to several limitations. First, the intervention was restricted to a single district in Egypt; therefore, the results may not be generalizable outside Minya. Additionally, the baseline surveys were conducted between May and July while the post-intervention surveys were conducted between October and December. Therefore, we cannot control for the temporal and seasonal effects on prescribing frequency and the influence of the survey timing on the pre- and post-intervention assessments. Similarly, although the same survey tool was used in the baseline and post-intervention surveys, the individual patients, pharmacists, and physicians surveyed in these two time periods differed due to the sampling approach, which introduces individual variability between the two populations. Therefore, we could conduct only descriptive comparisons and did not apply statistical methods to analyze the pre- and post-intervention results. Thus, we could not determine if the differences were due to the campaign alone or the influence of other confounding factors. However, since the campaign targeted all physicians and pharmacists in Minya, physicians and pharmacists were invited to participate in the pre- and post-intervention surveys and random samples of all healthcare clinics in Minya were taken for the patient surveys before and after the intervention. This approach allowed us to describe the pre- and post-intervention prescribing frequency and knowledge of antibiotic use throughout Minya. While the campaign targeted the Minya population broadly, we could not confirm that every individual was exposed to the intervention materials, a common limitation of public education campaigns. Finally, there is potential for social desirability bias in the self-reporting of prescribing practices by physicians and pharmacists as well as in responses to the beliefs and attitudes surveys.

## Conclusions

An educational intervention was implemented in Minya District, including a communication campaign, training of physicians and pharmacists, and provider outreach. Following the campaign, antibiotic prescribing for ARIs decreased and knowledge and attitude scores regarding appropriate use among physicians, pharmacists, and patients improved compared to the surveys conducted pre-intervention. Similar interventions may be effective in comparable settings where baseline antibiotic prescribing frequency is high and where patients are able to obtain antibiotics without prescriptions. Such interventions, resulting in reduced antibiotic prescribing, are needed to reduce selective pressures that are contributing to increasing antibiotic resistance worldwide and threatening global health security. The Ministry of Health and Population in Egypt is considering expansion of the campaign to more regions in Egypt to promote appropriate antibiotic use for ARIs using the Minya model as the basis for implementation.

## Abbreviations

ARIs: Acute respiratory infections
CDC: US Centers for Disease Control and Prevention
ENT: Otolaryngology
PHU: Primary healthcare units
